# Oxytocin reduces adipose tissue inflammation in obese mice

**DOI:** 10.1186/s12944-020-01364-x

**Published:** 2020-08-20

**Authors:** Angela Szeto, Monia Cecati, Raisa Ahmed, Philip M. McCabe, Armando J. Mendez

**Affiliations:** 1 Department of Psychology, University of Miami, PO Box 248185, Coral Gables, FL 33124 USA; 2 Department of Medicine and Diabetes Research Institute, University of Miami Miller School of Medicine, 1450 N.W. 10th Avenue, Miami, FL 33136 USA

**Keywords:** Adipose tissue, Anti-inflammation, Obesity, Oxytocin, Obese mouse model

## Abstract

**Background:**

Obesity and adipose tissue expansion is characterized by a chronic state of systemic inflammation that contributes to disease. The neuropeptide, oxytocin, working through its receptor has been shown to attenuate inflammation in sepsis, wound healing, and cardiovascular disease. The current study examined the effects of chronic oxytocin infusions on adipose tissue inflammation in a murine model of obesity, the leptin receptor-deficient (db/db) mouse.

**Methods:**

The effect of obesity on oxytocin receptor protein and mRNA expression in adipose tissue was evaluated by Western blotting and real-time polymerase chain reaction. Mice were implanted with osmotic minipumps filled with oxytocin or vehicle for 8 weeks. At study endpoint adipose tissue inflammation was assessed by measurement of cytokine and adipokine mRNA tissue levels, adipocyte size and macrophage infiltration via histopathology, and plasma levels of adiponectin and serum amyloid A as markers of systemic inflammation.

**Results:**

The expression of adipose tissue oxytocin receptor was increased in obese db/db mice compared to lean controls. In adipose tissue oxytocin infusion reduced adipocyte size, macrophage infiltration, IL-6 and TNFα mRNA expression, and increased the expression of the anti-inflammatory adipokine, adiponectin. In plasma, oxytocin infusion reduced the level of serum amyloid A, a marker of systemic inflammation, and increased circulating adiponectin.

**Conclusions:**

In an animal model of obesity and diabetes chronic oxytocin treatment led to a reduction in visceral adipose tissue inflammation and plasma markers of systemic inflammation, which may play a role in disease progression.

## Background

It has been suggested that obesity is characterized by a chronic state of systemic inflammation [1] that contributes to insulin resistance, dyslipidemia, diabetes and cardiovascular disease [2]. Obesity is accompanied by proliferation of adipose tissue [3] which consists of adipocytes, macrophages, fibroblasts, mesenchymal cells, pre-adipocytes, and endothelial cells [4]. Obesity-related inflammation is thought to be largely due to increased adipose tissue volume, infiltration of monocytes that differentiate into macrophages and express the classical M1 proinflammatory phenotype, and the subsequent secretion of pro-inflammatory cytokines and adipokines from adipocytes and macrophages [5]. It has been demonstrated that visceral adipose tissue, relative to subcutaneous fat, exhibits a greater inflammatory response as a consequence of obesity, evidenced by increased immune cell infiltration and cytokine production [6].

Oxytocin (OXT) is a neuropeptide that has been widely studied in terms of reproduction and social behavior, but more recently has gained attention for its anti-inflammatory properties. In wound healing and sepsis-induced animal models, subcutaneous injections of OXT were shown to diminish the immune and inflammatory response [7–9]. In our laboratory, we showed that macrophage OXT receptor (OXTR) expression is dramatically upregulated in response to inflammatory stimuli, suggesting that OXTR is an acute-phase protein that likely contributes to the anti-inflammatory actions of OXT [10]. We also demonstrated that chronic subcutaneous OXT infusions in apolipoprotein E knockout mice attenuated inflammatory atherosclerotic disease and the secretion of IL-6 from visceral adipose tissue [11]. Similarly, OXT infusions in the dyslipidemic Watanabe heritable hyperlipidemic rabbit slowed the progression of atherosclerosis and decreased plasma C-reactive protein levels, a biomarker of systemic inflammation [12]. Given that adipocytes and macrophages express OXTRs [10, 13], it is likely that adipose tissue represents an important target for the anti-inflammatory actions of OXT.

There is a growing literature that suggests OXT and its receptor may play an important role in regulating body weight and metabolism. For example, OXTR and OXT deficient mice exhibit late onset obesity and insulin resistance [14, 15]. In rodent models of obesity, chronic central or peripheral infusions of OXT have been shown to decrease weight gain [16–20] and improve insulin resistance [18, 20, 21]. These effects of OXT are at least partially due to central effects that decrease food consumption and increase energy expenditure [16–19].

The current study examined the effects of chronic OXT infusions on adipose tissue inflammation in a murine model of obesity and diabetes, the leptin receptor-deficient (db/db) mouse. It was hypothesized that the increased inflammation due to adipose tissue expansion in obesity would be attenuated by chronic OXT infusion. Inflammation was assessed by measuring macrophage infiltration into the visceral adipose tissue, as well as tissue expression and plasma levels of cytokines and adipokines. Our data show that OXTR expression is increased in adipose tissue of obese mice, and that OXT infusion attenuated adipose tissue inflammation. These data suggest a role for the OXT/OXTR pathway in peripheral tissue inflammation, and the potential use of OXT, or OXTR agonists, for the treatment of inflammatory disorders related to obesity.

## Methods

### Animal subjects

Mice were obtained from the Jackson Laboratory (Bar Harbor, ME, USA). Male C57BLKS/J Lepr^db−/−^ (db/db; Stock no. 000642) mice (leptin receptor deficient) and heterozygotes C57BLKS/J Lepr^db−/+^ (Stock no. 000662) having normal body weight, blood glucose, and plasma insulin served as lean controls. Although there are subtle differences in the magnitude of the inflammatory response among the various mouse models of obesity (e.g., DIO, ob/ob, db/db, *agouti* and *tubby*), it has been concluded that adipose tissue inflammation is a general phenomenon of the obese state [22] and therefore is present in all of these mouse models. The db/db mouse was chosen because it is a widely used model of obesity and diabetes [23, 24] without the concerns associated with the use of high fat diets [25]. The db/db mouse is characterized by an obese phenotype and exhibits many of the metabolic aberrations seen in human obesity, including insulin resistance, hyperglycemia and increased inflammation [23, 26, 27].

Although it is important to examine gender differences in obesity and inflammation, it has been shown that both OXT and OXTR expression vary significantly during the estrus cycle [28]. Therefore, in order to simplify the current study, we chose to focus on male mice, however, future studies in female are warranted.

Animals were housed 4 or 5 per cage at 22 °C on a 12:12 h day-night cycle and fed a normal chow diet (Rodent Chow 5001, LabDiet, Orlando, FL, USA) ad libitum. Mice were received at 6 weeks of age, were acclimated for 1 week, and then randomly assigned to a treatment group the following week and studies initiated when mice were 8 weeks of age. Ten mice per group were studied for the vehicle control and OXT treatment groups (see below), however, two mice in the control group did not complete the study (both due to surgical wounds from pump surgery that did not heal). Procedures for the care, use and euthanasia of experimental animals followed the protocols and regulations set forth by the Animal Care and Use Committee of the University of Miami and conformed to the Guide for the Care and Use of Laboratory Animals published by the US National Institutes of Health.

### Isolation of adipocytes and stromal vascular cells from adipose tissue

Epididymal adipose tissue was removed from lean control and db/db mice, minced, and digested using 2 mg/ml of collagenase type I (Sigma, St. Louis, MO, USA) in Hank’s balanced salt solution (HBSS) supplemented with 5.5 mM glucose, 15 mM HEPES, 100 mM sodium pyruvate, 1% bovine serum albumin, and 200 nM adenosine at 37 °C in a shaking water bath for 1 h. Tissue digests were filtered through 300 μm nylon cell strainers and adipocytes were separated by centrifugation (300 x g for 1 min), washed twice with PBS, and used for RNA extraction (below). The pelleted stromal vascular fraction (SVF) cells were treated with red blood cell lysis buffer for 5 min at room temperature, washed with PBS, centrifuged, and the SVF pellet was used for RNA extraction.

### Osmotic Minipumps and surgeries

Osmotic minipumps (Alzet model 1004, infusion rates of 0.11 μl/h, DURECT Corporation, Cupertino, CA, USA) were filled following manufacturer’s guidelines with 0.1 ml vehicle (50 mM sodium citrate, pH 4.0) or 1.6 mg/ml OXT (Bachem, Torrance, CA, USA) for an infusion rate of 4.22 μg/day/mouse. This dose of OXT was determined from previous work in our lab [11]. Mice were anesthetized with isoflurane (1–3% in 100% oxygen) and pumps were implanted subcutaneously in the mid-scapular region. Pump-exchange surgeries were performed once after 6 weeks.

### Necropsy

Mice were sacrificed by CO_2_ asphyxiation after 8 weeks of treatment (i.e., at 16 weeks of age). Blood was collected by cardiac puncture, transferred to a 1 ml Microvette tube containing EDTA (Sarstedt, Newton, NC, USA) and placed on ice until separation of plasma by centrifugation. Epididymal fat and internal organs were weighed. The fat was snap-frozen in liquid nitrogen or fixed, and frozen samples stored at − 80 °C.

### RT-PCR experiments to measure IL-6 and OXTR mRNA expression

RT-PCR was used to quantify cellular OXTR, and cytokine mRNA expression levels. Total RNA was isolated using RNeasy extraction kit (Qiagen, Valencia, CA, USA), then cDNA was synthesized after DNase I treatment using reagents from Applied Biosystems (Foster City, CA, USA) following manufacturer’s instructions.

Quantitative gene expression of mouse OXTR, IL-6, adiponectin, macrophage marker, F4/80, leptin, and TNF-α by RT-PCR was performed with the TaqMan gene expression assay. The following Applied Biosystems inventoried primers were used: mouse *Oxtr* (Mm01182684_m1), mouse *Il-6* (Mm00446190_m1), mouse adiponectin (*Adipoq*; Mm00456425_m1), *F4/80* (Mm00802529_m1), and *Tnf-α* (Mm00443260_g1). cDNA (50 ng) were amplified with TaqMan Universal PCR Master Mix and reactions were run using universal cycling conditions on an Applied Biosystems 7500 Real-Time PCR system. Samples were analyzed in triplicate. The ΔΔCT (threshold cycle) method was used to analyze changes in gene expression. Relative quantification (RQ) was expressed as the fold change compared to the appropriate control condition [29]. 18S rRNA was used as the endogenous RNA control. A non-template control was performed to ensure that there was no amplification of genomic DNA.

### Western blotting for OXTR

OXTR protein expression was examined in adipose tissue membrane fractions. Adipose tissue was homogenized in ice-cold lysis buffer (15 mM KCl, 1.5 mM MgCl_2_, 10 mM HEPES, 1 mM DTT, protease inhibitors), centrifuged at 1000 x g for 5 min at 4 °C, and OXTR enriched membrane fractions in the supernatant were isolated by centrifugation at 100,000 g × 60 min at 4 °C. The membrane pellet was solubilized in SDS lysis buffer (50 mM Tris, pH 8.6, 1% SDS). Protein was measured with BCA Protein Assay (Pierce, Rockford, IL, USA). OXTR protein expression was evaluated using previously described methods and using a specific polyclonal anti-rabbit OXTR (cat. no. ab181077; Abcam, Cambridge, MA, USA) validated against tissues from OXTR knockout mice [10]. Immunoreactive bands were detected with appropriate peroxidase conjugated secondary antibody for 1 h and then visualized with chemiluminescence (Clarity Max substrate, Bio-Rad Laboratories, Hercules, CA, USA). To normalize for protein loading, the membranes were stripped and re-probed with chicken anti-actin antibody (Cat. no. SAB3500350; Sigma, St. Louis, MO) or sodium/potassium ATPase (Cat. no. ab76020; Abcam, Cambridge, MA, USA).

### Quantification of adipocyte size

Formaldehyde-fixed paraffin embedded (FFPE) epididymal fat was sectioned at 4 μm and stained with hematoxylin and eosin. Five random fields from each section were imaged at 20X magnification. Adipocyte size (in μm^2^) was measured using the Adiposoft software program [30].

### Immunohistochemistry

FFPE epididymal fat sectioned at 4 μm were deparaffinized and antigen retrieval was carried out in Tris-EDTA buffer, pH 9.0, using a decloaking chamber. Endogenous peroxidase activity was quenched with 3% hydrogen peroxide and tissue sections were blocked with rabbit serum, incubated with F4/80 antibody (1100; BioRad, Cat. no., MCA497) or isotype control overnight. After incubation with secondary antibody, sections were exposed to avidin-biotin complex (Vectastain Elite ABC kit, Vector Laboratories, Burlington, CA, USA) then developed using 3,3′-diaminobenzidine (Vector Laboratories; Cat. no., 4100), and counterstained with Gill’s Hematoxylin (Vector Laboratories, Cat. no. H-3401).

For each slide, 8 images were captured with a color digital camera and a 10X objective. Images were processed using the HISTMATCH function of Matlab (MathWorks, Natick, MA, USA) to equalize the color histograms. Adjusted images were then used to quantify the immunoreactive areas with FIJI Image J [31] using color thresholding with the default setting, HSB color space and the same hue, saturation and brightness settings to select the color positive immunoreactive regions for all images. F4/80 immunoreactive regions were the average of 8 non-overlapping regions per slide for each tissue and expressed as the % of total area. Crown-like structures (areas where macrophages surround dying or dead adipocytes) were counted as previously described [6].

### Biomarker measurements

Plasma collected at endpoint was assayed for adiponectin, and serum amyloid A (SAA) using commercially available reagents (R&D Systems, Minneapolis, MN, USA; Millipore, Billerica, MA: Alpco, Salem, NH, USA, respectively). Plasma oxytocin was measured in unextracted plasma diluted 1:4 using the oxytocin chemiluminescent ELISA kit from Arbor Assay (Ann Arbor, MI, USA). Blood glucose was measured from the tail vein using OneTouch Ultra II glucometer and glucose strips (Lifescan Inc., Malvern, PA, USA).

### Lipolysis assay

Adipocytes were isolated from epididymal fat pads of three or four untreated db/db mice (approximately 16 to 20 weeks of age). Tissue was weighed, minced, and digested with collagenase as described above. Isolated adipocytes were incubated at 37 °C in Krebs-bicarbonate-HEPES buffer containing 3% fatty acid-free bovine serum albumin (Milllipore Sigma, Burlington, MA, USA), 10 mM sodium bicarbonate, 30 mM HEPES, 10 mM glucose and 500 nM adenosine [32] in the presence of OXT (0 to 1000 pM) and the amount of free fatty acid released in the medium and total triglyceride content of the adipocytes in each incubation were determined with commercial assays (NEFA assay and Triglyceride assay, Sekisui Diagnostics, Exton, PA, USA). Lipolysis was expressed as μmol of free fatty acid per μg of triglyceride.

### Statistical analyses

Data were shown to be normally distributed using the Shapiro-Wilk test and outliers were identified using the ROUT analysis in GraphPad Prism 8. Data are presented as means ± standard error of the mean (SEM). Results were compared by unpaired independent t-tests or ANOVA (one or two-way) with post hoc Bonferroni correction. An α-level of 0.05 was required for statistical significance.

## Results

### Expression of OXTR in adipose tissue

**Fig. 1:**
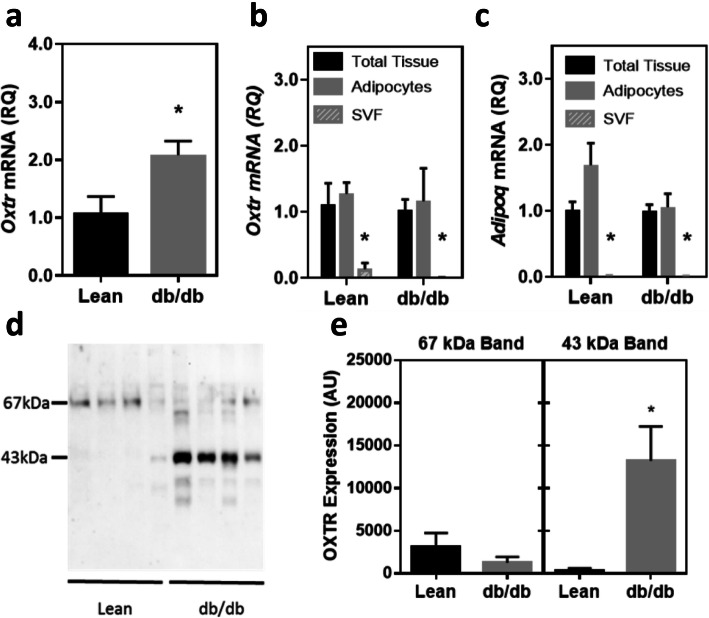
Obesity modulates OXTR expression in epididymal fat in lean and obese mice. OXTR mRNA expression relative to a control gene, 18S rRNA (Panel **a**, *n* = 9 per group; *p* < 0.05 comparing lean and db/db mice), Panels **b** and **c**; Distribution of *Oxtr* and adiponectin (*Adipoq*) mRNA, expression in total tissue, adipocyte fraction and stromal vascular fraction (SVF). Data were expressed relative to mRNA level in the total tissue for each respective group (*n* = 4 for lean, n = 4 for db/db). *Oxtr* and *Adipoq* expression were significantly greater in in the adipocyte fraction compared to the SVF fraction (both *p* < 0.05). Western Blot of OXTR from adipose tissue membrane fractions (Panel **d**). Molecular weight in kilodaltons (kDa) is indicated on the left. Densitometric quantification of OXTR protein for 67 and 43 kDa bands (Panel **e**) Data were expressed as mean ± SEM. * indicates *p* < 0.05.

Expression of *Oxtr* mRNA was evaluated in epididymal fat tissue from lean control and untreated db/db mice (Fig. 1a). Compared to lean controls there was a significant increase in *Oxtr* expression in the obese mice (*p* < 0.05). In order to evaluate the cell types that express *Oxtr* in adipose tissue, tissue was fractionated to obtain adipocytes and the stromal vascular fraction (SVF; Fig. 1b). In lean and obese mice, the vast majority of *Oxtr* mRNA expression was significantly greater in the adipocyte fraction compared to the SVF. Expression of adiponectin (*Adipoq*) mRNA, an adipocyte marker, was evaluated in the fractions to confirm separation of adipocytes from stromal cells (Fig. 1c). Expression of OXTR protein was evaluated in the same tissue (Fig. 1d and e), and was also increased in obese mice relative to controls. As previously shown in other tissues [10, 33] two immunoreactive OXTR bands were found in the fat tissue; an unglycosylated 43 kDa form and a mature glycosylated 67 kDa form. In the lean controls the predominant form of the protein was in the 67 kDa band. In the db/db mice there was a non-significant decrease in the 67 kDa band relative to the controls, but a dramatic 11-fold increase in the unglycosylated 43 kDa form of the protein.

### In vivo infusion of OXT in obese mice

**Fig. 2:**
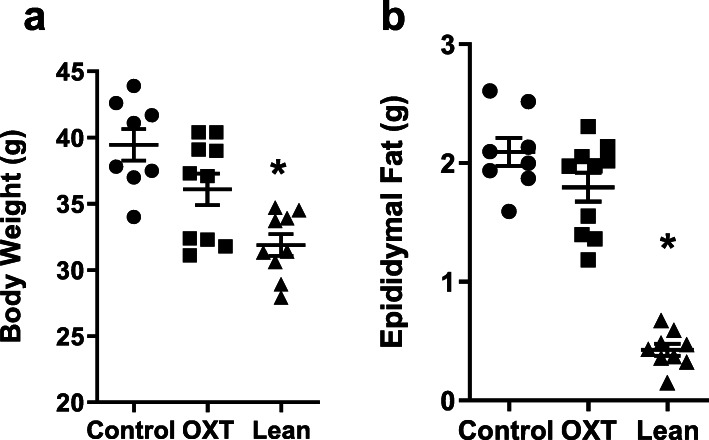
Body weight (Panel **a**) and epididymal fat weight (Panel **b**) in vehicle Control (*n* = 8) or OXT treated (*n* = 10) db/db mice after 8 weeks of treatment and in age matched lean control (n = 9) mice. Data were expressed as mean ± SEM. * indicates *p* < 0.05

**Fig. 3:**
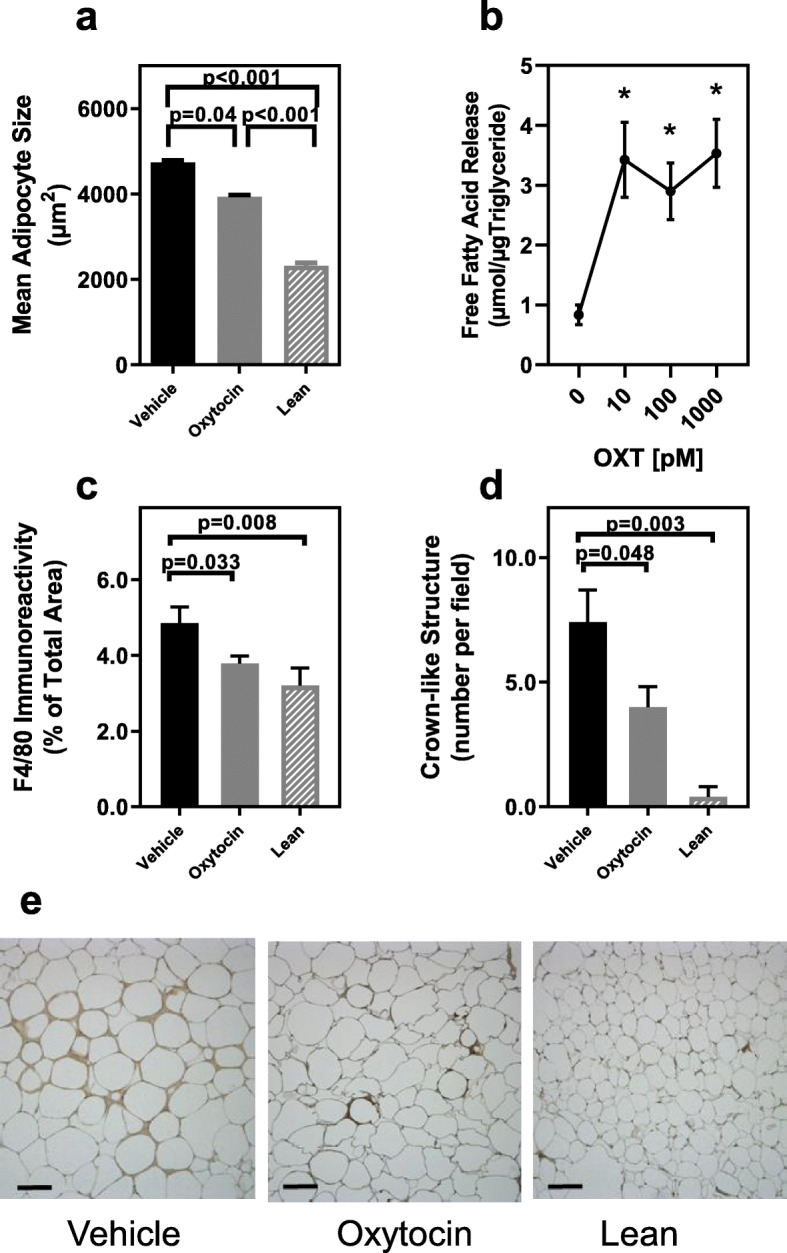
Effects of OXT infusion on adipocyte size, lipolysis and adipose tissue macrophage infiltration. (Panel **a**) Adipocyte size in epididymal tissues of Vehicle (n = 8) or OXT-treated db/db mice (n = 10) and lean control mice (*n* = 6). (Panel **b**) Lipolysis of triglycerides in isolated adipocytes from db/db mice incubated with increasing concentration of OXT for 30 min evaluated by release of free fatty acids. Adipocytes isolated from 3 or 4 mice were pooled and assayed in triplicate at each OXT dose. Data represent the mean for each dose from three separate experiments. OXT significantly increased lipolysis in a dose dependent manner; *p* = 0.003 for OXT dose and * indicates *p* < 0.01 compared to no oxytocin at all doses by post-hoc analysis (Panel **c**). Macrophage infiltration into adipose tissue of Vehicle (n = 8) or OXT-treated db/db mice (n = 10) and lean control mice (n = 6) assessed by immunohistochemistry for F4/80 antigen expression. (Panel **d**) Densities of crown-like structures in epididymal fat pads of db/db mice treated with vehicle (n = 8) or OXT (n = 9) and lean controls (n = 6) expressed as number per microscopic field (average of 5 fields per mouse tissue). (Panel **e**). Representative photomicrographs of F4/80 immunostained epididymal fat from vehicle and OXT treated db/db mice and lean control mice, bar = 50 μm. Data were expressed as mean ± SEM for each respective group.

OXT or vehicle was chronically subcutaneously infused via osmotic minipumps in db/db mice for 8 weeks. Plasma levels of OXT measured at the study endpoint were 79.4 ± 14.6 and 615 ± 103 for vehicle and OXT treated mice, respectively (*p* < 0.001). At study endpoint, vehicle control and OXT-treated mice had significantly greater body weight compared with lean controls (both *p* < 0.001), however, there was no significant differences between control and OXT-treated mice (*p* = 0.07). Fasting blood glucose was evaluated during the study and was similar in vehicle and OXT treated db/db mice throughout the study and no differences were observed in insulin sensitivity between groups in an insulin tolerance test (Supplemental Data, Figure 1). In addition, by the end of the study OXT-treatment did not affect liver, kidney, pancreas and spleen weights (data not shown). Epididymal fat mass was not different between vehicle control and OXT-treated db/db mice (*p* = 0.16) however, both showed significantly higher epididymal fat weight compared to lean control mice (both *p* < 0.001) (Fig. 2b). Chronic OXT infusion resulted in a 17% reduction in visceral adipocyte size (*p* = 0.04; Fig. 3a) in OXT-treated db/db mice compared with control db/db mice and both of these groups had larger adipocytes compared to the lean controls (*p* < 0.001). Consistent with this observation, in vitro studies from our lab using isolated adipocytes from db/db mice demonstrated that OXT can directly activate lipolysis of triglycerides (Fig. 3b). OXT infusion in db/db mice also resulted in a significant decrease in visceral fat macrophage infiltration compared to controls, as assessed by immunohistochemistry (Fig. 3c) and resulted in a decrease of crown-like structures (Fig. 3d, representative images are shown in Fig. 3e).

**Fig. 4:**
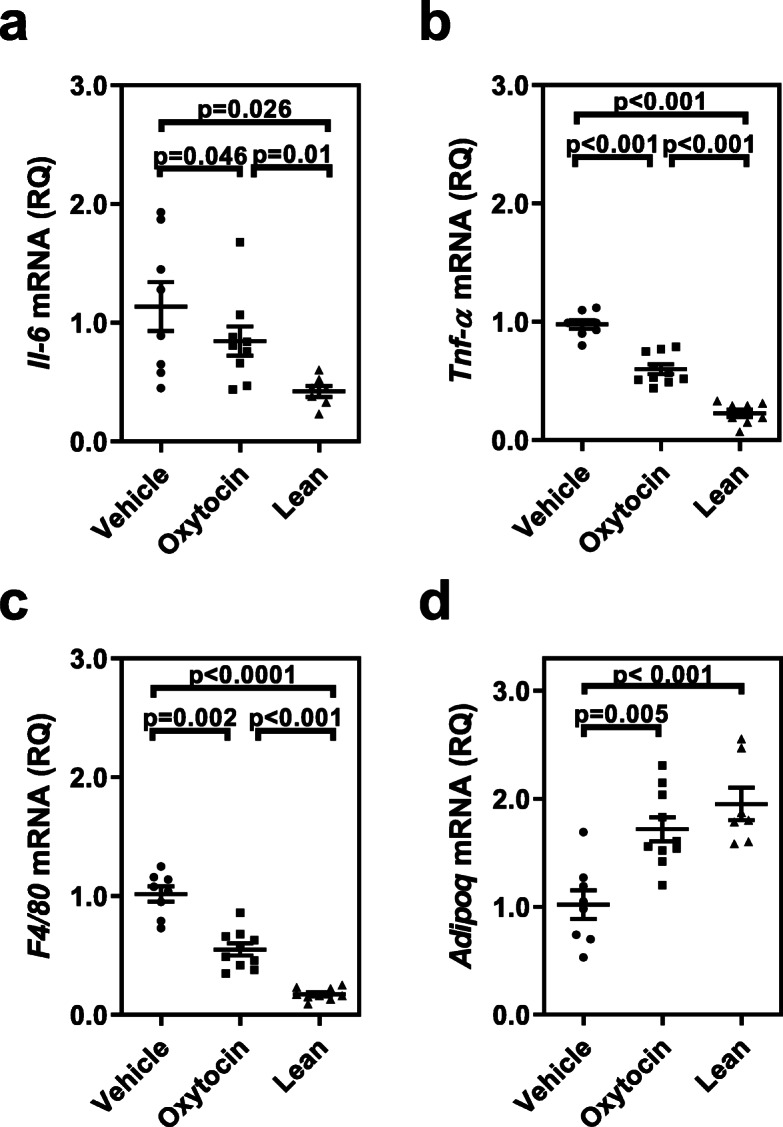
Adipose tissue mRNA expression of cytokines and adiponectin are altered in oxytocin-treated obese mice compared to vehicle control and lean mice. There was a significant decrease in mRNA expression for IL-6 (Panel **a**), TNF-α (Panel **b**), and in the macrophage marker, F4/80 (Panel C) relative to vehicle controls and lean mice. In contrast, oxytocin treatment led to an increase in the mRNA expression of adiponectin (Panel D), an anti-inflammatory adipokine. Data were expressed relative to 18S rRNA level for each respective group (n = 8 for vehicle, n = 10 for oxytocin-treated, and *n* = 7or 8 for lean)

Given these changes in adipose tissue phenotype, it was hypothesized that OXT infusion would alter adipokine expression, consistent with an anti-inflammatory action. Therefore, adipokine mRNA expression was evaluated in visceral adipose tissue from vehicle and OXT-treated obese mice, as well as lean controls. In general, OXT-treatment in the db/db mice shifted the adipokine expression in the direction of the lean controls (Fig. 4a-d). OXT infusion resulted in a significant decrease in mRNA expression of *Il-6* (Fig. 4a), *Tnf-α* (Fig. 4b), and macrophage marker, *F4/80* (Fig. 4c) compared to vehicle-controls. The lower F4/80 expression is consistent with the decreased macrophage infiltration observed in Fig. 3c. There was also a significant increase in mRNA expression of the metabolic modulator, adiponectin (Fig. 4d), in visceral fat compared to controls following OXT-treatment.

**Fig. 5:**
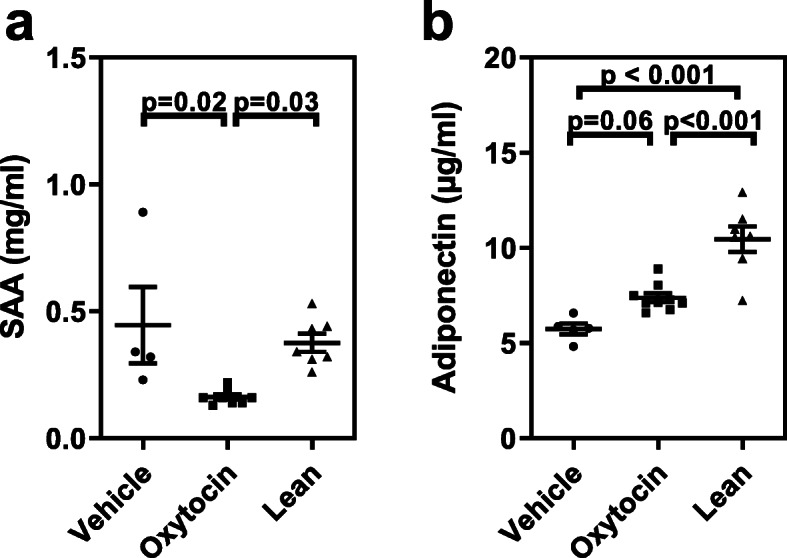
Oxytocin treatment altered plasma biomarkers of inflammation. (Panel **a**) Inflammatory biomarker, serum amyloid A (SAA) was attenuated in oxytocin-treated obese mice relative to vehicle controls. (Panel **b**) There was an increase in the anti-inflammatory plasma marker, adiponectin compared to vehicle controls. Data were expressed as mean ± SEM for each respective group (n = 4 for vehicle, n = 9 for oxytocin-treated, and n = 7 for lean)

In addition to visceral fat adipokines, plasma biomarkers related to systemic inflammation were evaluated. In particular, plasma serum amyloid A (SAA) was reduced in db/db mice following chronic OXT treatment, and in fact was significantly lower than both the vehicle-control obese mice and lean controls (Fig. 5a). For db/db mice, there was a trend for increased plasma adiponectin levels following OXT infusion (Fig. 5b; *p* = 0.06), which is consistent with the increased adiponectin mRNA expression observed in visceral fat tissue (Fig. 4d).

## Discussion

The major finding of this study is that in an animal model of obesity with hyperglycemia (the db/db mouse) chronic OXT treatment led to a reduction in visceral adipose tissue inflammation. Consistent with previously published work [21], OXT infusion reduced adipocyte size and macrophage infiltration into adipose tissue. In contrast to the prior study, these changes were observed in the absence of weight loss or alterations in glycemic control, suggesting that OXT works directly to suppress inflammation of adipose tissue rather than through reduction of adipose tissue mass or glycemic control regulation. This notion is further supported by a decrease in crown-like structures, reduced Il-6 and TNFα mRNA expression, and increased expression of the anti-inflammatory adipokine, adiponectin, in the adipose tissue. A possible mechanism by which OXT reduces adipocyte size is suggested by our data and that of others showing that OXT can directly stimulate triglyceride lipolysis [13, 16, 17]. Lastly, plasma SAA levels were decreased and plasma adiponectin levels were increased following OXT infusion, suggesting there was also a decrease in systemic inflammation.

Adipose tissue inflammation characterized by increased cytokine secretion and macrophage infiltration has been associated with obesity. Increased IL-6 secretion from adipocytes promotes adipose tissue macrophage accumulation [34]. TNF-α secretion from adipose tissue has been associated with insulin resistance that impairs glucose uptake, decreased fatty acid oxidation, increased adipose tissue mass [35, 36]. A variety of acute-phase response proteins including IL-6 and TNF-α have been shown to elevate plasma levels of SAA [37, 38]. Circulating levels of SAA are due to increased secretion by the liver, as well as adipocytes in response to inflammation [38] and serves as a stable biomarker of systemic inflammation. A beneficial effect of exogenous OXT administration may be a direct reduction of these inflammatory cytokine secretions from the adipose tissue thereby reducing macrophage infiltration and SAA protein expression. An additional benefit of OXT treatment is the increased expression of adiponectin, the anti-inflammatory effects of adiponectin are also associated with a healthier adipose tissue phenotype [39–41].

Obese db/db mice exhibited an increased expression of OXTR mRNA in adipose tissue compared to lean controls, which was previously reported in Zucker rats [42] and ob/ob mice [43]. This observation is consistent with prior work from our lab that demonstrated OXTR is upregulated in response to inflammation in macrophages [10]. These data suggest that activation of the OXTR by its ligand may be important in regulating adipose tissue inflammation and circulating inflammatory cytokines, thereby reducing systemic inflammation that results from obesity.

Two OXTR immunoreactive bands (presumably the N-glycosylated and unglycoslyated forms) were observed in adipose tissue, similar to previous findings in macrophage cells [44] and uterine tissues [45]. Compared to lean controls, obese mice exhibited an increase in adipose tissue OXTR mRNA, and an increase in protein expression associated with the unglycoslyated 43 kDa OXTR band. It is not clear why the two forms of the receptor exist in adipocytes or other cell types. It has been suggested that the glycosylation state of G protein-coupled receptors (GPCR) could influence transport to the plasma membrane [46]), ligand binding and affinity [33], degradation [47], and the formation of heterodimers with other GPCRs [48–50]. Clearly, further studies are needed to understand the functional significance of the two OXTR isoforms.

Previous studies have reported that peripheral OXT infusion significantly reduced body weight gain, adipose tissue mass and improved glycemic control [17–21, 43, 51]. These observations differ from the current study in that OXT infusion reduced adipose tissue inflammation without changes in body weight compared to control mice. These discrepancies could be explained by methodological differences among the studies, including variations among the animal models, OXT dosage, diet composition, duration of treatment, and age of the animals at study onset. For example, studies by Plante et. Al. [21] used the same mouse model but started treatment at an earlier age (4 week old mice compared to 8 week old mice in the current study) and for a longer treatment time (12 week vs. 8 week), used a lower OXT dose that increased plasma OXT levels by 2-fold (compared to ~ 8-fold) and had a final body weight of 52 g in the control mice compared to 40 g in this study. Studies by Altirriba et al. [43] in ob/ob mice showed that OXT treatment over two weeks reduced body weight gain compared to control mice but worsened glucose tolerance. Clearly, OXT treatment of obese mouse models can result in divergent effects. A key variable in the studies mentioned above are OXT dose and route of administration. Plante et al. infused 45 ng/day, Altirribba et al. used 5 μg/day and this study gave 4.2 μg/day all by continuous infusion. Such divergent doses are likely to affect receptor activity and regulation that may differ depending on the target tissue (reviewed in [52]) that may contribute to the observed differences across studies. Notwithstanding, several studies support the finding that peripheral OXT infusions attenuates adipose tissue and/or systemic inflammation [21, 43, 53].

OXT has gained attention as a putative treatment for obesity and type 2 diabetes (T2D) [54–57]. These studies propose that through central and peripheral mechanisms, OXT/OXTR may beneficially regulate glucose metabolism and insulin secretion. Given that systemic inflammation has been associated with the pathophysiology of obesity and T2D [58, 59], the anti-inflammatory actions of OXT/OXTR may also be important in attenuating disease progression.

### Study strengths and limitations

The strength of this study was the demonstration that oxytocin infusion reduced adipose tissue inflammation in an established mouse model of obesity, similar to the effects attributed to oxytocin in a variety of experimental inflammatory conditions [7, 11, 60–63] Limitations of this study include only the use of male mice so that sex differences, if any, were not evaluated. In addition, only a single dose of OXT that was continuously infused subcutaneously was evaluated, thus data on optimal dosage and routes of delivery should be evaluated in future studies. Additionally, given that OXT secretion occurs in a pulsatile manner (e.g., [64, 65]) effects of alternative dosing regimens should also be considered.

## Conclusions

In an animal model of obesity, chronic oxytocin treatment led to a reduction in visceral adipose tissue inflammation and plasma markers of systemic inflammation. The anti-inflammatory effects of OXT were observed in the absence of weight loss or changes in glycemic control, suggesting that OXT works directly to suppress inflammation of adipose tissue rather than through reduction in adipose tissue mass or glycemic regulation. Since obesity has been described as a state of chronic inflammation, dysfunction of the OXT/OXTR system may exacerbate inflammatory diseases/disorders related to obesity such as hypertension, diabetes, and heart disease. Conversely, the anti-inflammatory actions of OXT described in the current study may have utility as a therapeutic treatment to ameliorate some of the deleterious consequences associated with obesity [44, 66].

## Supplementary information

**Additional file 1: Figure 1S.** Fasting blood glucose and insulin tolerance testing in Control and Oxytocin treated db/db mice. **Table 1S.** Obesity modulates OXTR expression in epididymal fat in lean and obese mice.

## Data Availability

The datasets used and/or analyzed during the current study are available from the corresponding author on reasonable request.

## Abbreviations

OXT: Oxytocin
OXTR: Oxytocin receptor
db/db: Leptin receptor-deficient mouse
HBSS: Hank’s balanced salt solution
SVF: Stromal vascular fraction
PBS: Phosphate-buffered saline
EDTA: Ethylenediaminetetraacetic acid
DTT: Dithiothreitol
SDS: Sodium dodecyl sulfate
FFPE: Formaldehyde-fixed paraffin embedded
RQ: Relative quantification
SAA: Serum amyloid A
TNF-α: Tumor necrosis factor
IL-6: Interleukin 6
GPCR: G protein-coupled receptor
T2D: Type 2 diabetes
NEFA: Non-esterified fatty acids
SEM: Standard error of the mean
