# Supplementary material for: Oxytocin reduces adipose tissue inflammation in obese mice
Source: Lipids Health Dis. 2020 Aug 20;19:188. doi: 10.1186/s12944-020-01364-x (PMC7441653; doi:10.1186/s12944-020-01364-x)
Supplement: Supplementary file 1 — Additional file 1: Figure 1S. Fasting blood glucose and insulin tolerance testing in Control and Oxytocin treated db/db mice. Table 1S. Obesity modulates OXTR expression in epididymal fat in lean and obese mice. [file 12944_2020_1364_MOESM1_ESM.docx]

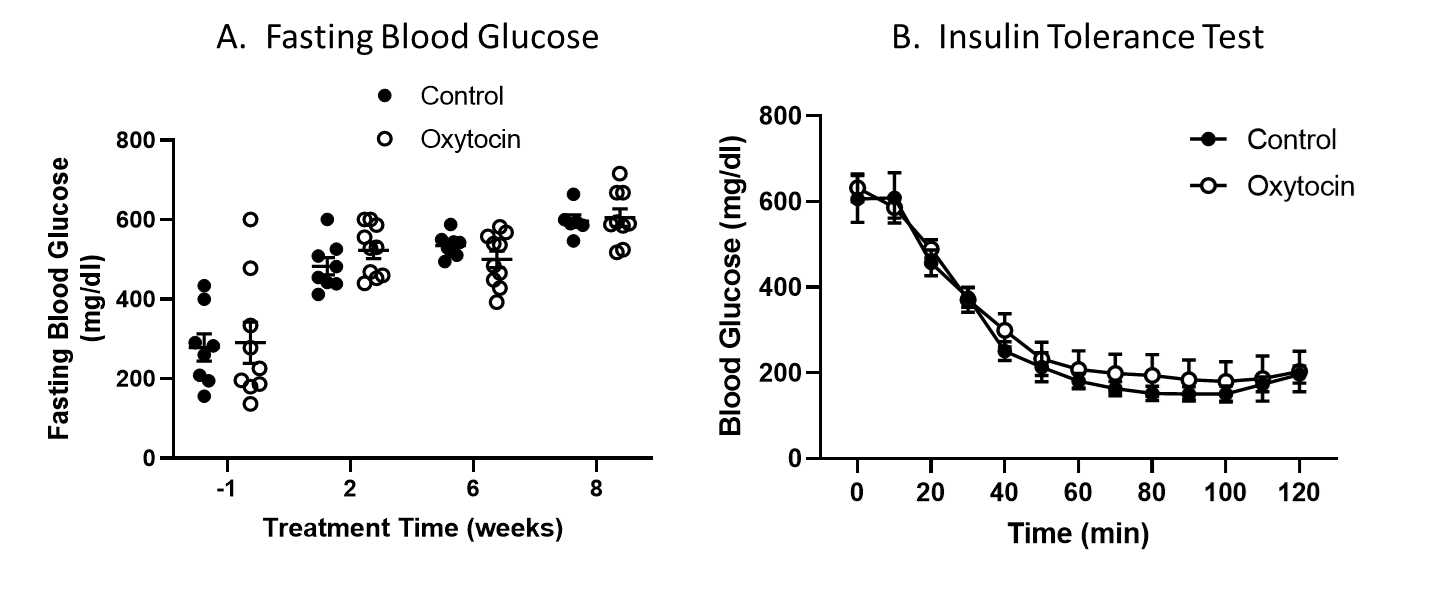


Figure 1S. (A) Blood glucose was measured after a 6 hour fast in vehicle or OXT treated db/db mice at the indicated study time points relative to pump implantation. (B) Insulin tolerance testing was performed in vehicle Control (n=8) or Oxytocin (=10) treated db/db mice after 8 weeks of treatment. After a 6 hour fast, mice were injected with 3 U insulin/kg body weight and blood glucose was measured at the indicated time points. Data are means and SEM and there were no statistical differences between groups.

| **VALUES FOR DATA IN FIGURE 1** | | | | |
| --- | --- | --- | --- | --- |
| **Figure 1A** | OXTR mRNA (RQ) | | | |
|  | Lean (n=9) | | db/db (n=9) | |
| Unfractionated Adipose Tissue | 1.09 | ± 0.28 | 2.09 | ± 0.24 |
| **Figure 1B** | OXTR mRNA (RQ) | | | |
|  | Lean (n=4) | | db/db (n=4) | |
| Unfractionated Adipose Tissue | 1.11 | ± 0.32 | 1.02 | ± 0.14 |
| Adipocyte Fraction | 1.27 | ± 0.17 | 1.17 | ± 0.49 |
| Stromal Vascular Fraction | 0.14 | ± 0.09 | 0.02 | ± 0.00 |
| **Figure 1C** | ADIPO mRNA (RQ) | | | |
|  | Lean (n=4) | | db/db (n=4) | |
| Unfractionated Adipose Tissue | 1.01 | ± 0.13 | 1.00 | ± 0.09 |
| Adipocyte Fraction | 1.69 | ± 0.33 | 1.06 | ± 0.20 |
| Stromal Vascular Fraction | 0.02 | ± 0.00 | 0.02 | ± 0.00 |
| **Figure 1E** | OXTR Protein Expression (AU) | | | |
|  | Lean (n=4) | | db/db (n=4) | |
| 67 kDa Band | 3062 | ± 850 | 1259 | ± 350 |
| 43 kDa Band | 329 | ± 142 | 13220 | ± 4017 |

Table 1S. Obesity modulates OXTR expression in epididymal fat in lean and obese mice. Refer to Figure 1 for details
